# Integrating sequencing methods with machine learning for antimicrobial susceptibility testing in pediatric infections: current advances and future insights

**DOI:** 10.3389/fmicb.2025.1528696

**Published:** 2025-03-05

**Authors:** Zhuan Zou, Fajuan Tang, Lina Qiao, Sisi Wang, Haiyang Zhang

**Affiliations:** ^1^Department of Emergency, West China Second University Hospital, Sichuan University, Chengdu, China; ^2^Key Laboratory of Birth Defects and Related Diseases of Women and Children, Sichuan University, Ministry of Education, Chengdu, China; ^3^Department of Pediatrics, West China Second University Hospital, Sichuan University, Chengdu, China

**Keywords:** next-generation sequencing, Oxford Nanopore Technologies, machine learning, antimicrobial resistance, pediatrics

## Abstract

Antimicrobial resistance (AMR) presents a critical challenge in clinical settings, particularly among pediatric patients with life-threatening conditions such as sepsis, meningitis, and neonatal infections. The increasing prevalence of multi- and pan-resistant pathogens is strongly associated with adverse clinical outcomes. Recent technological advances in sequencing methods, including metagenomic next-generation sequencing (mNGS), Oxford Nanopore Technologies (ONT), and targeted sequencing (TS), have significantly enhanced the detection of both pathogens and their associated resistance genes. However, discrepancies between resistance gene detection and antimicrobial susceptibility testing (AST) often hinder the direct clinical application of sequencing results. These inconsistencies may arise from factors such as genetic mutations or variants in resistance genes, differences in the phenotypic expression of resistance, and the influence of environmental conditions on resistance levels, which can lead to variations in the observed resistance patterns. Machine learning (ML) provides a promising solution by integrating large-scale resistance data with sequencing outcomes, enabling more accurate predictions of pathogen drug susceptibility. This review explores the application of sequencing technologies and ML in the context of pediatric infections, with a focus on their potential to track the evolution of resistance genes and predict antibiotic susceptibility. The goal of this review is to promote the incorporation of ML-based predictions into clinical practice, thereby improving the management of AMR in pediatric populations.

## 1 Introduction

Infectious diseases pose a significant threat to global health, placing immense strain on public health systems worldwide. Infants and children, due to the immaturity of their immune systems, are particularly vulnerable to these threats (Zhou et al., 2024). During early development, children are exposed to a wide range of viruses, which often lead to bacterial co-infections, resulting in sepsis rates that are higher than those observed in adults (Kissoon and Carapetis, 2015). Extended hospital stays are known to facilitate the development of resistance among common pathogens against empirical treatment regimens, further contributing to increased mortality from sepsis and meningitis in pediatric patients (Williams et al., 2024). The rise of antimicrobial resistance (AMR) represents a critical global public health crisis, particularly affecting pediatric populations. A study in China found that 71.76% of pediatric subjects carried multi-drug-resistant (MDR) gram-negative bacteria, with only 8.23% showing susceptibility to the tested antibiotics (Patil et al., 2019; Xu et al., 2023). Similar trends have been documented globally (Kim et al., 2022; Nisa et al., 2022). The rapid escalation of AMR in children demands urgent development of novel strategies to combat MDR infections and reduce unnecessary pediatric mortality (Medernach and Latania, 2018). This growing threat highlights the pressing need for rapid antimicrobial susceptibility testing (AST) and vigilant monitoring of AMR through comprehensive laboratory data.

Recent advances in sequencing technologies have greatly enhanced the diagnosis of infectious diseases, particularly through techniques such as metagenomic next-generation sequencing (mNGS), Oxford Nanopore Technologies (ONT), and targeted sequencing (TS). These technologies are invaluable for diagnosing pediatric respiratory, central nervous system, and bloodstream infections due to their ability to rapidly and accurately detect pathogens in clinical samples (Horiba et al., 2018; Okumura et al., 2023; Zeggeren et al., 2021). They also enable the timely identification of antibiotic resistance genes (ARGs), often within 24–48 h, improving clinical outcomes. mNGS provides a broad and unbiased analysis by detecting all microbial species in clinical samples. It involves fragmenting microbial genomes into small pieces (50–500 bp) and sequencing them against both local and public reference databases to identify pathogens and ARGs (Chiu and Miller, 2019). In contrast, TS targets a specific set of microbial species by using predefined primers to capture and sequence ARGs. ONT, on the other hand, facilitates the single-pass sequencing of long DNA fragments (> 10 kbp) via electrical signal detection, providing detailed insights into mutations and genetic variations, which is particularly useful for tracking ARGs (Wang C. et al., 2021). Although the results of AST can be influenced by factors such as gene regulation, environmental conditions, and microbial interactions (e.g., horizontal gene transfer), there is substantial evidence correlating the presence of AMR genes with drug-resistant phenotypes in microorganisms (Zhang et al., 2022).

The growth of whole-genome sequencing (WGS) data, combined with clinical AST results and advancements in AMR databases, has significantly boosted the application of machine learning (ML) in predicting AMR phenotypes in pediatric infections. When trained on large, diverse datasets, ML models can achieve high prediction sensitivities, reaching up to 96.3% by analyzing AMR determinants (Girgis et al., 2023; Yang et al., 2019). However, while most studies have focused on using ML to interpret AST outcomes derived from WGS data, there remains a notable gap in research exploring the integration of ML with other sequencing technologies commonly used in clinical pathogen diagnostics (Gao W. et al., 2024). This gap underscores the need for further investigation into how these technologies, especially when combined with ML, can improve diagnostic accuracy and therapeutic decision-making in pediatric settings.

Machine learning has already found widespread application in pediatric disease management, including diagnosis, screening, risk stratification, prognosis, and outcome prediction. It has been particularly useful in identifying serious bacterial infections in children (Clarke et al., 2022; Fenta et al., 2024; Lee et al., 2022). This review examines the role of sequencing technologies in clinical practice, focusing on their application in pediatric care for detecting AMR in pathogens, monitoring the evolution of ARGs, and tracking resistance gene dynamics. Additionally, the review addresses the challenges associated with developing ML models that can accurately predict AST outcomes. The primary objective of this study is to highlight the impact of sequencing technologies on the management of infections and antibiotic sensitivity in pediatric populations. Furthermore, it explores the potential for integrating ML-driven predictions of drug sensitivity into clinical workflows, with the aim of improving patient care and treatment outcomes.

## 2 Confronting microbial resistance in pediatric infectious diseases

### 2.1 Prevalence and AMR

The physiological and anatomical characteristics of children differ markedly from those of adults, particularly due to their immature physiological functions and underdeveloped immune systems. These differences make pediatric populations particularly susceptible to a wide range of infectious diseases that affect multiple organ systems. In pediatric patients, prevalent infections encompass respiratory conditions like pneumonia and bronchitis; gastrointestinal infections such as gastroenteritis and Helicobacter pylori-related illnesses; neurological disorders including encephalitis and meningitis; as well as integumentary and soft tissue infections, sepsis, fungal infections, tuberculosis, and parasitic diseases. Among these, respiratory infections are the most prevalent, affecting more than 40% of children (Pscheidt et al., 2021). While many children experience mild symptoms, the unique aspects of their growth and development can sometimes lead to severe illness. Additionally, the spectrum of pathogens responsible for these infections varies by geographic region. For example, *Escherichia coli* (*E. coli*) is frequently isolated in neonates, school-aged children, and adolescents, while *Haemophilus influenzae* is more commonly found in infants. *Streptococcus pneumoniae* primarily affects toddlers and preschoolers (Wu et al., 2023). Additionally, respiratory epidemics in children are often driven by pathogens such as influenza virus, rhinovirus, Mycoplasma pneumoniae, and adenovirus.

Bacterial meningitis and meningoencephalitis are rapidly progressing, severe infections that contribute significantly to the high rates of morbidity and mortality in pediatric populations. These conditions are most prevalent in children under 5 years of age and typically spread through hematogenous routes, accounting for up to 90% of cases. Notably, infants and young children represent two-thirds of these cases, with fatality rates ranging from 5% to 10% (Li et al., 2018).

Invasive fungal diseases (IFD) are also a significant concern in pediatric populations, particularly within intensive care settings. Aspergillus species are the leading causative agents of IFD in children, accounting for approximately 40% of these infections, with *Aspergillus fumigatus* being the most commonly isolated species (Burgos et al., 2008). Other common agents include *Trichoderma* spp. and *Fusarium* spp. (Pana et al., 2017). Invasive candidiasis is frequently observed in children with bloodstream infections (Baalaaji, 2022; McCarty et al., 2021). Additionally, other atypical fungi, such as *Cryptococcus neoformans*, *Pneumocystis jirovecii*, and *Talaromyces marneffei*, pose significant clinical risks. Diagnosing IFD in children is particularly challenging due to the low rates of positive fungal cultures and the atypical clinical presentations of these infections (Bandalizadeh et al., 2020; García-Moreno et al., 2020; Guo et al., 2019; Zeng et al., 2021). The presence of co-infections with viral and bacterial pathogens—especially multidrug-resistant organisms—further complicates diagnosis and contributes to prolonged hospital stays, as well as increased morbidity and mortality (Donnelly et al., 2020).

The early identification of pathogens is often uncertain, which can lead to the unnecessary or inappropriate use of antibiotics, sometimes without microbiological confirmation (Thaulow et al., 2019). In pediatric patients, resistant infections may arise from exposure to drug-resistant strains or the emergence of resistance in previously susceptible strains during hospitalization. Neonates, whose immune systems are immature and whose resistance is low, are especially vulnerable to resistant pathogens. Several factors exacerbate this risk, including preterm birth, low birth weight, vertical transmission from mother to child, perinatal infections, and the inappropriate use of antibiotics.

The increased prescription of antimicrobial agents during the COVID-19 pandemic has likely contributed to the accelerated emergence of multidrug-resistant (MDR) bacteria. Notably, there has been a rise in the detection of drug-resistant *Staphylococcus aureus* (*S. aureus*) in both pediatric and maternity wards (Pinheiro et al., 2023). School-aged children are particularly burdened by invasive *S. aureus* isolates, while premature and very low-birth-weight infants are at an increased risk for early-onset sepsis caused by isolates resistant to both ampicillin and gentamicin. Additionally, invasive *Streptococcus pneumoniae* isolates in children show higher resistance rates to penicillin and other antibiotics, including erythromycin, clindamycin, and mebendazole/sulfamethoxazole, compared to adults (Thaulow et al., 2021). Alarmingly, resistance rates among coagulase-negative staphylococci in children with bloodstream infections have reached 91%, and *E. coli* isolates exhibit significant resistance to cefotaxime, with 84% of isolates resistant (Sajedi Moghaddam et al., 2024).

**FIGURE 1:**
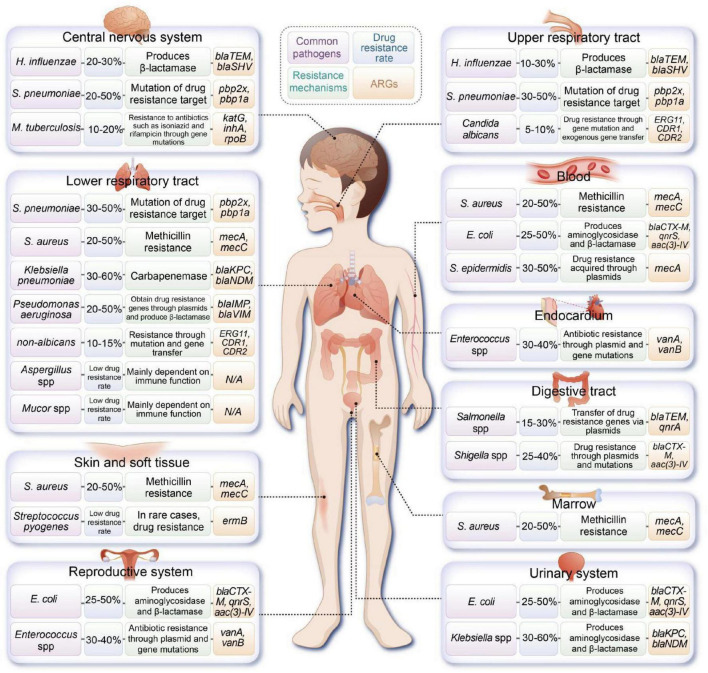
Overview of common pathogens implicated in pediatric infections at various anatomical sites, along with their documented resistance mechanisms and associated antibiotic resistance genes (ARGs). (*H. influenzae, Haemophilus influenzae; S. pneumoniae, Streptococcus pneumoniae; M. tuberculosis, Mycobacterium tuberculosis; S. aureus, Staphylococcus aureus; E. coli, Escherichia coli*).

Moreover, multidrug-resistant *Klebsiella pneumoniae* (*K. pneumoniae*) is an emerging contributor to sepsis in pediatric patients, with high resistance levels to beta-lactam antibiotics (Vijayakumar et al., 2024). The incidence of highly virulent *K. pneumoniae* in the pediatric population is rapidly increasing, underscoring the critical nature of the AMR crisis that is now affecting children (Li et al., 2021 ^)^. Figure 1 illustrates the common pathogens and associated ARGs found in various sites of infection in pediatric patients.

### 2.2 Limitations of conventional pathogen identification methods

Conventional methods for pathogen diagnosis encompass pathogen isolation and culture, microscopic examination, immunological assays, and polymerase chain reaction (PCR). Microbial cultures can be classified into aerobic, anaerobic, and specialized cultures, each requiring substantial sample volumes. Specialized cultures refer to those designed for isolating specific or fastidious pathogens that may require particular environmental conditions or selective media. Examples include mycobacterial cultures for tuberculosis, fungal cultures for detecting mycoses, and cultures for detecting specific pathogens like *Brucella* or *Legionella*. However, acquiring sufficient samples from pediatric patients, particularly neonates, presents significant challenges.

The blood culture procedures in children are inherently more complex than those in adults, often necessitating multiple blood draws to ensure accurate identification. Collecting blood from pediatric patients can be particularly difficult, as younger children may not comprehend the necessity of bilateral double sampling, potentially compromising the effectiveness of culture methods. Consequently, the sensitivity and specificity of these traditional diagnostic approaches are relatively low, making it difficult to detect mixed infections and leading to possible missed diagnoses of various infectious diseases. Immunological methods that detect pathogen antigens, metabolites, or antibodies in serum facilitate the identification of new or secondary infections and allow for the monitoring of disease progression. However, the underdeveloped immune systems of children may delay antibody production, which can result in the missed detection of pathogens.

In recent years, advanced molecular testing technologies, such as the FilmArray^®^ Respiratory Panel, have significantly improved the detection rates of pathogens in lower respiratory tract infections. These technologies have proven particularly effective in pediatric populations, achieving detection rates between 70% and 80% for various pathogens. This advancement not only reduces reliance on antimicrobial drugs but also shortens their duration of use, consequently diminishing the need for additional diagnostic procedures such as chest radiographs, thereby leading to more efficient utilization of medical resources (Kitano et al., 2020; Subramony et al., 2016). Nonetheless, multiplex PCR technology has limitations in throughput, typically detecting only a restricted number of pathogenic microorganisms. Some kits are also limited to specific sample types, which may restrict their applicability across a broader range of specimens. Additionally, when microbial load is low—often the case with pediatric samples—there is a risk of missed detections, potentially resulting in overlooked diagnoses. The introduction of matrix-assisted laser desorption/ionization time-of-flight mass spectrometry (MALDI-TOF) has expanded the capabilities for identifying clinical microorganisms, including a wider array of bacterial and fungal species. This technology has reduced reporting times to 18–24 h, significantly enhancing the diagnostic timeline. However, it is crucial to consider that culture results can be influenced by multiple factors, including the use of antimicrobial agents, pathogen load, and the selection of culture media (Martín et al., 2018).

## 3 Advances in antibiotic resistance detection technologies

### 3.1 Conventional methods for detecting antibiotic resistance

Bacterial drug resistance has emerged as a critical global threat. In 2011, the World Health Organization (WHO) issued an urgent alert on World Health Day, stating, “Curbing drug resistance—if we don’t act today, we won’t have a drug tomorrow.” This warning is particularly pertinent in pediatric care, where the developing organs of children are more vulnerable to the adverse effects of antimicrobial overuse, especially with broad-spectrum antibacterial agents (Zhang et al., 2018). Data from the China Children’s Multi-center Antimicrobial Drug Application Monitoring Network indicates a troubling trend: the proportion of broad-spectrum antimicrobial usage has remained elevated in recent years, with no significant decline observed (Wang C. et al., 2021). This sustained overuse exacerbates the critical issue of pathogenic microorganism resistance among children.

To mitigate this crisis and slow the emergence of resistance, thereby reducing the risk of therapeutic failure, a targeted approach is essential. Antibiotic therapy should only be initiated after determining the resistance profile of the causative pathogen. This strategy ensures that treatment is both effective and minimizes the contribution to the development of further resistance.

Pathogen drug-sensitivity testing is a fundamental aspect of modern antimicrobial stewardship. This testing includes both phenotypic and genotypic assessments of drug resistance. Among these, AST is particularly prominent. AST typically employs methods such as broth dilution, gradient diffusion, and disc diffusion, conducted after pathogen culture. Results are interpreted according to the guidelines established by the United States Committee for Clinical Laboratory Standards (CLSI) in 2022. While AST has a long history and generally provides reliable and cost-effective outcomes, its effectiveness is contingent upon successful culture, and results can be influenced by multiple factors, including the type of culture medium, bacterial inoculum size, drug concentration, incubation duration, and temperature (Jenkins and Schuetz, 2012).

Moreover, AST results do not always predict therapeutic success. This discrepancy can occur because *in vitro* testing conditions may not accurately reflect *in vivo* environments. Consequently, *in vitro* AST outcomes may not correlate well with the treatment of certain chronic diseases ^(^ Flynn et al., 2020; Somayaji et al., 2019). Therefore, it is crucial to interpret drug sensitivity test results within the broader context of the patient’s clinical condition.

### 3.2 mNGS

In 2019, a review in JCM underscored the significant utility of WGS and mNGS in predicting bacterial AMR. These advanced techniques offer comprehensive and systematic insights into ARGs through high-throughput sequencing (Boolchandani et al., 2019). mNGS, in particular, enables the detection of all microorganisms in clinical samples, facilitating the identification of AMR genes. Following nucleic acid extraction, library construction, and sequencing, the obtained sequences are compared with public or in-house resistance gene databases to pinpoint ARGs (Diao et al., 2022). Studies have demonstrated a reasonable alignment between mNGS results for AMR detection and clinical AST outcomes. For instance, Gan et al. (2024) utilized mNGS to predict AMR in pediatric patients with severe pneumonia, observing superior sensitivity in predicting carbapenem resistance compared to penicillins and cephalosporins. This variability in predictive performance highlights the dependence on the pathogen and antibiotic in question.

Moreover, mNGS is increasingly being applied in the analysis of microbial communities and functional genomic research pertinent to pediatric infections. This technique allows for the detection of both up-regulation and down-regulation of ARGs functions, thereby aiding in the early management of AMR in pediatric populations (Xu et al., 2023). Furthermore, ML models have been developed to predict antibiotic susceptibilities for pathogens identified through mNGS. A study employed mNGS to ascertain the antibiotic susceptibility profiles of *Acinetobacter baumannii*, creating an ML model that achieved a diagnostic accuracy exceeding 96.5% in both retrospective and prospective validations (Hu et al., 2023). Another investigation used mNGS data to detect *K. pneumoniae* in pediatrics and predict its AMR profile with an ML-based approach, resulting in an area under the curve (AUC) value greater than 0.9, with prediction accuracies ranging from 88.76% to 96.26% (Xu Y. et al., 2024).

### 3.3 Third-generation sequencing

The emergence of third-generation sequencing technologies, such as ONT or long-read sequencing methods, has been a game-changer in genome assembly. These technologies provide exceptionally high-quality data, allowing for direct detection of epigenetic modifications on native DNA or RNA, and offer a portable solution for genomic analysis (Wang C. et al., 2021). ONT sequencing excels in identifying specific species and strains within samples due to the mappable nature of long reads. This capability provides a more accurate assessment of microbiota composition compared to traditional methods that rely on 16S rRNA and DNA amplicons (Van Dijk et al., 2018). Remarkably, ONT can identify pathogens in under 10 min and detect ARGs within an hour, a significant acceleration over conventional culturing methods, which typically require 2–3 days, plus additional time for AST results (Taxt et al., 2020). This technology has demonstrated remarkable performance in diagnosing childhood infections (Trotter et al., 2019).

Beyond rapid, real-time pathogen detection, ONT offers sensitivity and accuracy comparable to mNGS and targeted next-generation sequencing (tNGS), such as those offered by Illumina, in detecting pediatric RNA viruses. It has also been extensively applied in diagnosing tuberculosis (TB) in children. When, combined with 16S rDNA sequencing, ONT enhances sensitivity and lowers the detection limit for pneumonia infections in pediatric patients (Chen et al., 2023; Wang X. et al., 2023). In the realm of ARGs detection, long-read nanopore sequencing excels by identifying the presence or absence of ARGs, determining the phenotype of AMR, differentiating between ARG subtypes, and pinpointing specific pathogens. This capability is a significant advantage over conventional methods. Additionally, ONT provides a distinct benefit in accurately identifying low-abundance plasmid-mediated AMR, which often eludes detection by traditional techniques (Liu et al., 2023). The rapid, application of real-time genomics holds substantial promise for advancing clinical decision-making and improving patient outcomes (Sauerborn et al., 2024).

### 3.4 TS

Targeted sequencing offers versatility across various sequencing platforms, with tNGS being a particularly effective method. tNGS identifies pathogens by employing specific primers designed for pathogen detection, AMR and virulence genes (Sauerborn et al., 2024). As the range of these specific primers expands, so does the capacity to detect a broader spectrum of pathogens and AMR genes, scaling from dozens to hundreds, and even thousands in some cases (Goh, 2019). The sequencing data generated by these genetic panels significantly enhance bacterial read-length sequences and genome coverage (Allicock et al., 2018; Tang et al., 2024). tNGS has proven particularly effective in detecting *Mycobacterium tuberculosis complex* (MTBC) infections and associated ARGs (Murphy et al., 2023). It reliably predicts AMR in MTBC directly from clinical specimens or cultures, providing essential information for the timely and appropriate treatment of drug-resistant tuberculosis. While WHO-recommended rapid diagnostic technologies can detect rifampicin resistance, they often fall short in identifying other forms of tuberculosis AMR, such as isoniazid resistance or resistance to second-line drugs (Farhat et al., 2024). In contrast, tNGS can detect resistance to over a dozen anti-tuberculosis drugs in a single test, including both conventional and new drug types, with a specificity of ≥ 95% for anti-tuberculosis AMR (Kamb Li et al., 2021).

Targeted next-generation sequencing has rapidly become the preferred method for diagnosing TB pathogens and AMR in pediatric patients, demonstrating superior sensitivity compared to the Xpert test for detecting pulmonary tuberculosis in children (Zheng et al., 2024). Integrating tNGS with ML modeling further enhances the diagnostic accuracy for TB infection and AMR, with predictive sensitivities exceeding 95% in blood and cerebrospinal fluid samples (Cryptic Consortium, 2024a; Shaikh and Rodrigues, 2023). Moreover, tNGS maintains high sensitivity (70.8%–95.0%) in samples with low pathogen loads, such as blood and cerebrospinal fluid, and performs well in detecting ARGs in respiratory infections in children, showing good concordance with erythromycin and tetracycline resistance (Chen et al., 2024; Lin et al., 2023).

Nanopore Targeted Sequencing (NTS), powered by third-generation sequencing technology, offers a comprehensive approach to AMR detection, transcending the limitations of hotspot mutations and significantly reducing sequencing time. NTS has demonstrated a high level of concordance with AST results, particularly in detecting MTBC-AMR (Gao W. et al., 2024). This method enables the precise identification of a broad spectrum of resistance gene profiles, including MDR and extensively drug-resistant strains (Tang et al., 2024). Compared to single-target assays, NTS provides a simpler setup and delivers results more rapidly than WGS and phenotypic AST (Allicock et al., 2018; Sonntag et al., 2024).

**FIGURE 2:**
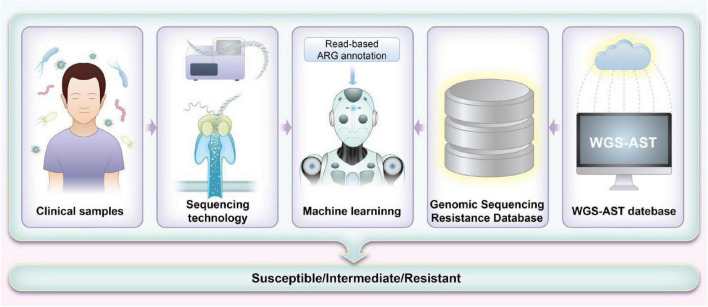
Workflow for machine learning (ML)-based prediction of antimicrobial resistance (AMR) using sequencing methods. The process starts with collecting and preparing whole-genome sequencing (WGS) data and corresponding antimicrobial susceptibility testing (AST) results. Low-quality data are filtered to ensure accuracy. Genomic sequences are aligned with the Comprehensive Antibiotic Resistance Database (CARD) database to identify potential resistance markers. These markers train ML models, which are evaluated for their predictive accuracy. Features are weighted based on significance, and models are refined and validated with clinical samples. The results are then used to improve clinical decision-making and guide antibiotic stewardship.

Each sequencing method provides unique insights that form the foundation for integrating ML approaches aimed at predicting antibiotic susceptibility in pediatric patients. The detailed genomic information obtained through these sequencing methods, when combined with advanced ML algorithms, significantly enhances our ability to predict and customize antibiotic treatments. By leveraging the comprehensive data from sequencing, ML models can improve the accuracy of susceptibility predictions and guide more personalized therapeutic strategies. Table 1 summarizes the sequencing methods employed for detecting ARGs in pediatrics, while Figure 2 illustrates how ML techniques are applied to predict AMR based on sequencing data. Together, these advancements represent a pivotal shift toward more precise and effective management of AMR in pediatric infections.

**TABLE 1:** Sequencing methods used for antibiotic resistance genes (ARGs) detection in pediatrics.

Type	Detection technology	Detection type	Culture needed?	Advantages	Limitations	References
Traditional AST detection methods	Disk. diffusion method	Phenotype	Yes	1. Simple and does not require specialized equipment 2. Simultaneous testing for multiple antibiotics 3. With standardized protocol	1. Time-consuming 2. Unable to get MIC value 3. Dependence on subjective interpretation 4. Affected by temperature, humidity, pH and other factors	Bonev et al., 2008; Weinstein and Lewis, 2020
	Agar dilution and broth dilution methods	Phenotype	Yes	1. Widely recognized and used, With standardized protocol 2. MIC value can be obtained 3. Cost minimization, and convenience	1. Time-consuming 2. Inefficient, only one antibiotic can be tested at a time 3. Effects of emulsifiers and solvents	Golus et al., 2016; Intra et al., 2019
	Antimicrobial gradient diffusion test	Phenotype	Yes	1. Convenient and rapid 2. Excellent reproducibility 3. MIC value can be obtained 4. Simultaneous detection of multiple antibiotics	1. Not applicable to other types of antimicrobials 2. Subject to subjective interpretation	Gupta et al., 2015; Jeverica et al., 2017
Mass spectrometry	MALDI-TOF	Genotype	Yes	1. Less time consumption and accuracy 2. Easy to interpretation 3. Drug resistance genes in plasmids can be detect	1. Equipment is expensive 2. Specialized person needed	Florio et al., 2020; Singhal et al., 2015
Sequencing technologies	WGS	Genotype	Yes	1. Detect the full spectrum of drug-resistant genes 2. Tracking new emerging mutations and emerging drug resistance 3. High throughput 4. High sensitivity and specificity	1. Time-consuming 2. Costly 3. Complex bioinformatics analysis	Almaghrabi et al., 2024; Monk, 2019
	mNGS	Genotype	No	1. Simultaneous detection of pathogens and export of resistance genes 2. Cultured free 3. High throughput 4. Rapid	1. Influenced by human genetic background 2. Costly 3. Insufficient sequencing depth	(Hu et al., 2023; Xu Y. et al., 2024)
	ONT	Genotype	No	1. Long Sequence 2. Cultured free 3. More rapid than mNGS 4. Drug resistance genes in plasmids can be detect	1. Influenced by human genetic background 2. Costly 3. Prone to sequencing errors	Nisa et al., 2022; Van Dijk et al., 2018
	TS	Genotype	No	1. Rapid 2. High throughput 3. Simultaneous detection of pathogens and export of resistance genes 4. Independent of human genetic background	1. Require prior knowledge of target genes 2. Challenges of aerosol pollution 3. Inability to identify emerging variants	Tang et al., 2024; Murphy et al., 2023

MALDI-TOF, matrix-assisted laser desorption/ionization time of flight mass apectrometry; WGS, whole-genome sequencing; mNGS, metagenomic next-generation sequencing; ONT, Oxford Nanopore Technologies; TS, targeted sequencing; AMR, antimicrobial resistance.

## 4 Challenges in sequencing methods for predicting drug sensitivity

The role of ML in predicting pathogen drug sensitivity has gained considerable attention in recent years. Despite the inconsistent correlation between genotypic and phenotypic resistance detection, the clinical application of sequencing-based resistance genotype identification encounters significant challenges. Integrating this methodology into clinical practice remains difficult, hindering its potential to facilitate more effective antibiotic utilization. However, ML offers a promising solution by leveraging extensive datasets of resistance genes and their phenotypic relationships. This approach enables the accurate prediction of resistance levels, including the estimation of minimal inhibitory concentrations (MIC) for resistant, intermediate, and susceptible profiles.

In pediatrics, the broad range of clinical presentations, atypical symptomatology, and rapid disease progression add significant complexity to diagnosis and treatment (Guo et al., 2020). Children are particularly vulnerable to AMR, and the challenges associated with obtaining high-quality clinical samples further hinder pathogen detection and AST methods. These factors highlight the urgent need for advanced and precise diagnostic approaches (Wu et al., 2024). Additionally, many pediatric patients receive antibiotics prior to hospital admission, which compromises the reliability of culture-based diagnostic methods, particularly for respiratory pathogens. Therefore, improving the enrichment of pathogen-derived nucleic acids in clinical samples is critical for optimizing sequencing accuracy, thereby introducing another layer of complexity to the diagnostic process (Wilson et al., 2019).

### 4.1 Challenges in mNGS

In principle, ML can predict antimicrobial drug susceptibility by analyzing ARGs identified through sequencing (Hyun et al., 2023). However, the effectiveness of ML models is contingent on both the sequencing technology employed and the quality of the data obtained. Most studies currently utilize AST by combining WGS of cultured bacterial strains with ML algorithms. This approach is advantageous due to the comprehensive data it generates, including information on alleles, non-allelic variants, and genes that may not directly contribute to AMR but are relevant to ARG prediction. Despite its potential, several challenges remain that need to be addressed before these technologies can be routinely used in clinical practice.

One significant challenge is the time required for microbial cultivation, which typically ranges from 2 to 4 days, depending on the complexity of the organism. In the case of *Mycobacterium tuberculosis* (MTB), this process can extend from 2 to 8 weeks. This delay in obtaining cultured samples can result in prolonged diagnostic turnaround times, which is especially concerning in pediatric patients who may require rapid intervention. Furthermore, the large volume of data generated by WGS requires extensive bioinformatics analysis, which adds to the overall processing time (Su et al., 2019). Another challenge arises with mNGS, a promising approach that is prone to complexities related to experimental conditions. These include control over environmental microbial contamination, the initial DNA input in samples, sequencing depth, and the analytical capabilities required to handle vast amounts of data. Additionally, mNGS results are susceptible to false positives due to background noise, which includes genetic material from human hosts and environmental organisms. This contamination complicates the interpretation of results, making it difficult to determine whether the identified microorganism is the true causative agent of infection. Such issues become more pronounced in cases where the read depth for detected pathogens is low, when results do not align with the clinical presentation, or when rare or atypical pathogens are detected. These challenges, while significant, underscore the importance of continued technological improvements in sequencing methods and ML algorithms, as well as the need for more robust bioinformatics tools and clinical workflows. Addressing these obstacles will ultimately facilitate the broader implementation of sequencing-based AST and ML approaches in pediatric infection management, enabling faster, more accurate diagnoses and more effective treatments.

The data preprocessing stage of mNGS can be facilitated using software tools such as Trimmomatic v0.39, which removes low-quality sequence fragments and trims sequences with inadequate length. Transcriptomic technologies, such as RNA sequencing, provide valuable insights into bacterial gene expression, which can help bridge gaps in our understanding of AMR. RNA sequencing can detect ARGs directly in clinical samples without the need for prior culture; however, its effectiveness is dependent on sample quality. For instance, obtaining high-quality sputum samples from children under 5 years of age is often challenging (Shaikh and Rodrigues, 2023). Studies on pediatric patients with severe pneumonia have shown that the average genome coverage for pathogen detection is often low, with the highest recorded coverage at 42.25%. While mNGS demonstrates good sensitivity and specificity for detecting carbapenem resistance, sensitivity is considerably reduced for other resistance mechanisms (Gan et al., 2024; Su et al., 2021). The integration of ML methods has led to successful predictions of AST outcomes in several studies (Lüftinger et al., 2023; Xu Y. et al., 2024). However, since mNGS relies on random fragment sequencing, it may fail to cover important genomic regions, which can hinder accurate microbial typing. This results in insufficient data on species and ARGs, particularly for known chromosomal mutations that are detectable but may be missed due to sequencing limitations. Additionally, mutations in resistance-related genes, such as point mutations involved in resistance mechanisms, are often challenging to detect (Asante and Osei Sekyere, 2019; Hadjadj et al., 2019). False-negative results inconsistent with AST outcomes are also a concern (Pailhoriès et al., 2022). Moreover, plasmid-mediated resistance mechanisms are difficult to detect using mNGS. Both WGS and mNGS based on short-read sequencing are prone to missing key genetic information due to incomplete coverage of critical genomic sites. To mitigate this, multiple software programs, such as Readfq V82 for quality assessment and tools like fastp and SPAdes for sequence assembly, are required to screen and optimize gene sequences (Chen et al., 2018). Despite these efforts, incorrect assembly can lead to erroneous results, and even with quality control improvements, the resolution of ARGs remains challenging (Gao W. et al., 2024). Furthermore, while efforts are made to reduce the human genetic background in clinical samples, a significant amount of human DNA often persists, which can contribute to detection failures. In pediatrics, samples with low microbial content may result in non-uniform genome coverage or insufficient sequencing depth, undermining the reliability of ARG detection (Ruppé et al., 2022). Additionally, mNGS often cannot localize ARGs to specific pathogens, which compromises the accuracy of drug sensitivity predictions.

### 4.2 Challenges in third-generation sequencing

Third-generation sequencing technology can simultaneously detect multiple resistance genes and all resistance loci, providing more comprehensive information on drug resistance. Overall categorical agreement of 95% for penicillins, 82.4% for cephalosporins, 76.7% for carbapenems, 86.9% for fluoroquinolones, and 96.2% for aminoglycosides in a study (Weinmaier et al., 2023). However, Third-generation sequencing is currently overpriced, the higher error rates associated with ONT and the difficulty in resolving homopolymer sequences pose significant challenges. These can affect the accuracy of AMR determinations in children, where precise genomic information is paramount. Additionally, the choice of extraction kit can influence the variability of third-generation sequencing results, affecting factors such as reporting time, sequencing yield, and accuracy. Moreover, *Pseudomonas* species are common contaminants of ONT kits and reagents, and the presence of nucleic acids from these organisms may interfere with the accuracy of drug resistance predictions (Cryptic Consortium, 2024a). In addition, the rate of false positives for drug-resistant genes was higher in third-generation sequencing than in second-generation sequencing because of sequencing or assembly problems. Variability in the sequencing platform’s electrical signal stability could introduce biases, influencing both the interpretation of genomic data and the efficacy of subsequent ML models in predicting resistance patterns (Istace et al., 2017). Conversely, ONT offer promising advancements in pathogen AMR detection through its capacity for long-read sequencing. This technique preserves crucial mutation sites within ARGs, vital for tracking resistance patterns in pediatric pathogens.

### 4.3 Challenges in TS

The TS approach enhances the detection of AMR in pediatrics by minimizing the interference of host DNA, thereby increasing the assay’s sensitivity compared to mNGS (Datar et al., 2021). mNGS and tNGS have comparable detection rates of pathogenic microorganisms in children, both for respiratory and tissue samples (Gaston et al., 2022; Miao et al., 2018). Their application in detecting drug resistance genes in clinical samples has become widely adopted. Lin et al. (2023) utilized tNGS to identify a total of 58 ARGs associated with tetracyclines, macrolides-lincosamides-streptozotocin, beta-lactams, sulfonamides, and aminoglycosides in 19 out of 25 pediatric patients. The concordance rates between tNGS results and those from traditional AST for erythromycin, tetracycline, penicillin, and sulfonamides were 89.5%, 79.0%, 36.8%, and 42.1%, respectively (Banerjee and Patel, 2023). Nonetheless, TS is susceptible to common challenges associated with PCR, including non-specific amplification and aerosol contamination. Additionally, issues such as limited primer specificity can result in false-positive or false-negative outcomes. Moreover, the reliance on meticulously designed probes and PCR primers restricts genomic coverage, particularly failing to detect sequences outside the predefined panel, which may overlook emerging resistance genes critical in pediatrics. It facilitates the resolution of plasmid sequences, enabling a more detailed investigation of HGT of ARGs (Boolchandani et al., 2019).

### 4.4 Impact of sequencing parameters on outcomes

The effectiveness of sequencing test kits in predicting antibiotic sensitivity in pediatric infections is significantly influenced by their quality. Robust assays necessitate stringent quality metrics, including a Q30 score of at least 75% for accurate base detection, a minimum of 50,000 original reads to ensure adequate sequence data, and thresholds of 200 and 3,000 reads for internal parameter amplification and pathogenicity target regions, respectively (Kastanis et al., 2019). However, several inherent challenges related to sequencing technology remain. These challenges include difficulties in tracing the origins of drug-resistant genes to specific microorganisms, the need for clinical correlation to interpret the significance of identified bacterial species, and the complex task of distinguishing between infectious and colonizing microorganisms, which often requires additional clinical context. Addressing these challenges is crucial for enhancing the reliability of sequencing methods in predicting antibiotic sensitivity, particularly in the pediatric population, where atypical symptoms and rapid disease progression complicate diagnostic processes (Zhu et al., 2023).

## 5 The crucial role of comprehensive and up-to-date genetic data in drug resistance

Large-scale public datasets are crucial for ML models aimed at predicting AST outcomes. These databases must be both comprehensive and regularly updated to improve the accuracy of drug susceptibility predictions, especially for newly approved antibiotics (Cryptic Consortium, 2024a). The Comprehensive Antibiotic Resistance Database (CARD) exemplifies this necessity for regular updates, with its latest version, 3.2.4, released monthly (Alcock et al., 2023). However, the performance of ML models can be compromised if critical resistance features of certain strains are overlooked during feature selection (Tian et al., 2024). While databases like ResFinder have been updated to version 4.0, others, including the Antibiotic Resistance Genes Database (ARDB) and Antibiotic Resistance Gene-ANNOTation (ARG-ANNOT), are no longer maintained (Xu Y. et al., 2024). The reliability of these reference datasets is crucial for establishing baseline trends and patterns.

Currently, genomic information, including resistance data, is unevenly distributed across public databases, exhibiting a bias toward data from developed regions such as North America and Europe. Although contributions from developing countries, particularly Asian nations like China, are increasing, they still lag behind their Western counterparts (Alygizakis et al., 2024). The NORMAN ARB&ARG database, established by the NORMAN Association, incorporates information on antibiotic-resistant bacteria and ARGs from studies conducted in China and Nepal (Porse et al., 2020). Furthermore, China has developed DRESIS, the world’s first comprehensive database on AMR, which covers a broad spectrum of disease categories and resistance mechanisms (Papp and Solymosi, 2022). The continuous incorporation of high-quality microbial and ARGs data, alongside timely updates to these databases, is essential for advancing the clinical applicability of drug susceptibility predictions. Further details regarding AMR gene databases can be found in Table 2.

**TABLE 2:** Overview of databases for antibiotic resistance genes (ARGs).

Databases name	Update frequency	Data types	Clinical applications	Database features	References
CARD	Regular updates, typically quarterly	Gene sequences, resistance mechanisms, gene annotations, metadata	Identification and analysis of ARGs and mechanisms.	Comprehensive coverage of resistance genes, detailed annotations, and integrated tools for resistance prediction.	Alcock et al., 2023; Liu and Pop, 2009
ARDB	Periodic updates, less frequent	Gene sequences, resistance profiles, functional annotations	Gene identification and characterization of resistance in various pathogens.	Includes data on resistance genes, mutations, and mechanisms; supports genomic searches.	Xu Y. et al., 2024; Bortolaia et al., 2020
ResFinder	Frequent updates, typically monthly	Gene sequences, resistance profiles, mutation data	Detection of ARGs in sequencing data, especially for clinical diagnostics.	Focuses on ARGs in pathogenic bacteria, includes high-throughput sequencing data.	Florensa et al., 2022; Gupta et al., 2014
ARG-ANNOT	Regular updates, typically quarterly	Gene sequences, functional annotations, taxonomic data	Annotation of ARGs in metagenomic and genomic sequences.	Annotates ARGs with functional and taxonomic information, supports various sequencing data formats.	Johnson et al., 2022
ARGO	Less frequent updates, annual	Gene ontology terms, functional annotations, gene sequences	Functional classification and understanding of ARGs.	Provides ontology-based classification, integrating gene function and resistance mechanisms.	Bush, 2023
Lahey list of β-lactamases	Periodic updates, biannual	Gene sequences, β-lactamase classifications, enzyme characteristics	Identification and characterization of β-lactamase genes and their variants.	Comprehensive list of β-lactamases, including detailed information on gene variants and enzyme properties.	Naas et al., 2017
BLDB	Regular updates, typically quarterly	Gene sequences, enzyme activities, resistance profiles	Tracking and analysis of β-lactamase genes and their resistance profiles.	Extensive database of β-lactamases with information on gene sequences, enzyme activities, and clinical relevance.	Prasanna and Niranjan, 2019
TBDReaMDB	Periodic updates, less frequent	Gene sequences, novel β-lactamase variants, resistance data	Research and development of β-lactamase-related treatments and diagnostics.	Focuses on β-lactamase gene research, including novel variants and their implications for drug resistance.	Gibson et al., 2015
Resfams	Regular updates, typically annual	Gene family classifications, functional annotations, sequence data	Classification and functional annotation of ARGs families.	Provides family-based classification and functional annotation of ARGs across diverse species.	Pei et al., 2024

ARGs, antibiotic resistance genes; CARD, Comprehensive Antibiotic Resistance Database; ARDB, Antibiotic Resistance Genes Database; ARG-ANNOT, Antibiotic Resistance Gene-ANNOTation; ARGO, Antibiotic Resistance Genes Online; BLDB, β-lactamase database; TBDReaMDB, tuberculosis AMR mutation database.

A major limitation of these databases is their inability to capture unknown resistance gene mechanisms. Additionally, the coexistence of multiple resistance mechanisms can influence the final AMR phenotype, potentially compromising the performance of ML models (Papp and Solymosi, 2022). Furthermore, the lack of standardization across databases adds another layer of complexity, as inconsistencies and variations in data across databases may affect the accuracy and reliability of ML model predictions.

## 6 Key considerations in applying machine learning for predicting antibiotic susceptibility in pediatrics

### 6.1 Genomic datasets and feature extraction

Machine learning in microbiology, particularly for predicting AST from sequencing data, has significant potential to improve clinical decision-making in pediatric settings. Table 3 summarizes existing studies that integrate ML with sequencing approaches for AMR analysis in pediatric populations. Among the various ML techniques employed, Random Forest (RF) and Logistic Regression (LR) models appear to be the most commonly used across pediatric studies, reflecting their broad applicability and robustness in handling complex datasets such as whole genome sequencing (WGS) and NGS data. These models are frequently applied due to their relatively straightforward implementation and strong predictive performance, particularly in contexts like bloodstream infections and urinary tract infections. Conversely, more complex models, such as Bayesian networks, Support Vector Machines (SVM), and Naïve Bayes, are used less often, potentially due to their greater computational demands or limited adaptation to the diverse nature of pediatric infections. Despite the promise of integrating ML with sequencing technologies, challenges remain in the accurate prediction of pediatric antimicrobial resistance, including issues related to the limitations of sequencing technologies like mNGS, tNGS, ONT, and TNS, and the need for further optimization in ML model selection and integration for pediatric-specific infections.

**TABLE 3:** Summary of pediatric studies integrating machine learning with sequencing approaches for antimicrobial resistance analysis.

References	Study date	Age range	Sequencing approach employed	ML approach used	Key findings/notes
Lee et al., 2022	2022	0–6 years	WGS	RF, CLR	Identified SBI in pediatric patients
McAdams et al., 2022	2022	Neonate (< 28 days after birth)	WGS	Supervised learning, regression models	Investigated neonatal morbidities, including sepsis, bronchopulmonary dysplasia, and retinopathy of prematurity
Sick-Samuels et al., 2020	2020	0–18 years	WGS	Decision tree	Focused on bloodstream infections caused by multidrug-resistant gram-negative organisms
Bagnasco et al., 2022	2022	0–19 years	WGS	LR	Explored urinary tract infections in pediatric patients
Xuemei et al., 2024	2024	> 28 days after birth to 18 years	WGS	LR, RF, XGB, and BP	Investigated children with severe infections
Frange et al., 2012	2012	0–18 years	WGS	SVM	Estimated the potential benefits of CCR5-antagonist therapy in pediatric patients
Yap et al., 2016	2016	Neonate (< 28 days after birth)	tNGS	RF	Focused on intestinal carriage of multidrug-resistant gram-negative bacteria in neonates
Karsies et al., 2018	2018	> 28 days after birth to 14 years	WGS	Multivariable regression	Investigated gram-negative bacilli with high antibiotic resistance potential
Rahman et al., 2018	2018	0–3 months	mNGS	RF	Identified influential ARGs in the infant gut microbiome
Gasparrini et al., 2019	2019	0–21 months	mNGS	RF	Analyzed persistent metagenomic signatures in pediatric patients
Wu et al., 2020	2020	0–17 years	WGS	Bayesian networks	Predicted causative pathogens in children with osteomyelitis
Lu et al., 2022	2022	0–18 years	WGS	LR	Investigated urinary tract infections in Chinese pediatric patients
Sung et al., 2022	2022	0–18 years	tNGS	RF	Studied *Mycoplasma pneumoniae* pneumonia in children
Tsurumi et al., 2023	2023	0–16 years	tNGS	LASSO	Focused on infections in children with burns
Hoffer et al., 2024	2024	2–17 years	tNGS	SVM	Investigated acute *Streptococcal* Pharyngitis in children
Xing et al., 2024	2024	0–18 years	mNGS	LASSO	Studied childhood infectious meningitis and encephalitis
Özdede et al., 2024	2024	0–18 years	Pangenome analysis	Naïve bayes	Analyzed carbapenem-resistant *Acinetobacter baumannii* infections in pediatric patients

RF, random forest; LR, logistic regression; SBI, serious bacterial infection; DT, decision tree; XGB, extreme gradient boosting tree; BP, backpropagation neural network; SVM, support vector machine; tNGS, targeted Sequencing; mNGS, metagenomic sequencing; LASSO, least absolute shrinkage and selection operator.

The “garbage in, garbage out” principle is a fundamental aspect of ML, emphasizing the importance of high-quality input data. This principle is especially crucial in pediatrics for several reasons. First, the diversity within pediatric populations, ranging from neonates to adolescents, introduces significant variability in physiological and pathological features at each developmental stage. For example, neonates and adolescents have distinct immune systems, metabolic rates, and drug responses (Simon et al., 2015). If these age-related differences are not adequately reflected in the input data, the model’s predictions may be inaccurate or even harmful to certain age groups (Wiens and Shenoy, 2018). Second, data collection in pediatric settings presents unique challenges, such as children’s limited ability to accurately express symptoms, necessitating reliance on parental reports (Varni et al., 1999). Additionally, pediatric datasets are typically smaller, and data quality and completeness may be compromised. Although large datasets are not always essential, genomic datasets should be sufficiently large to ensure robust model training. However, larger datasets increase processing time, which could be a limiting factor in pediatric contexts. Pediatric ML models should ideally be developed from datasets where the sample size exceeds the number of features to ensure class balance and model validity. Low-quality or incomplete data can lead to models learning incorrect or unreliable information, compromising the model’s performance and reliability (Raghupathi and Raghupathi, 2014b). Third, pediatric data collection and use are governed by strict ethical and privacy regulations to protect confidentiality and security. These regulations can limit data access and sharing, further restricting dataset size and diversity (Olteanu et al., 2019). However, dataset size and sample selection bias often hinder the effectiveness of ML models in clinical settings (Goto et al., 2019). Thus, ensuring high-quality and representative input data is crucial for developing and applying ML models in pediatric contexts.

Feature extraction and selection are crucial for the predictive accuracy and efficiency of ML models. Inaccurate feature extraction can lead to model failure. ARGs are key genetic determinants of antimicrobial resistance and are often conserved across various species (Macesic et al., 2020). High-dimensional feature vectors may negatively impact ML performance and increase execution time (Wang C. C. et al., 2023). Algorithms such as RF and LASSO have built-in feature selection capabilities, which help in removing low-abundance or low-prevalence features. Prodigal, a gene prediction tool based on open reading frames, provides detailed feature predictions that enhance the filtering process (Hyatt et al., 2010). Additionally, k-mer length can be used as an input feature for ML models to predict the MIC. Feature selection methods apply equally to the input dataset, which is crucial for ensuring the relevance of features used in ML models. In metagenomic sequencing, commonly employed techniques include NGS and ONT. Raw data undergo preliminary processing before feature selection, which involves the removal of low-quality reads and adapter sequences using tools such as FastQC, fastp, and NanoFilt. It is also essential to eliminate human nucleic acids using tools like Bowtie2 and Kraken. High-quality reads are then assembled into genomic contigs using assembly software like SPAdes, and subsequently mapped to an ARGs database using BLASTN. The ARGs database is pre-organized based on the research objectives to identify target ARGs and their single nucleotide polymorphisms (SNPs), insertions, and deletions. Alternatively, features can be selected based on gene presence/absence and gene variants for further analysis. However, it is vital to ensure that the selected ARGs are specific to the pathogens of interest in the current research (Bortolaia et al., 2020; Hu et al., 2023; Xu Y. et al., 2024).

Optimizing features, especially those highly correlated with drug susceptibility phenotypes, can significantly improve model performance (Sarker, 2021). In pediatric antibiotic susceptibility assessments, consideration should not only be given to the presence of known resistance genes but also to genomic mutations relative to reference genomes. Studies on pediatric pathogen drug sensitivity have shown that the AUC for predicting antimicrobial resistance increases as the length of the compared sequence fragments increases, particularly in MTB (Aytan-Aktug et al., 2021). However, reliable sequence length requirements for accurate prediction have yet to be established. Precise identification of resistance-related genes and ensuring high-quality sequencing are crucial to improving the accuracy of ML models in managing pediatric infectious diseases.

### 6.2 ML algorithms

The performance of ML algorithms during training is influenced by various factors, one of the most important being the structure and order of the input dataset. This raises concerns about the reproducibility of the results: will the outcomes remain consistent if the algorithm is trained on the same data but with different input sequences? To ensure robust and reproducible model performance, it is critical to implement strategies such as random data splitting and cross-validation during training. These techniques help mitigate biases introduced by the sequence of the input data.

Furthermore, ML algorithms require large, diverse, and well-annotated datasets to train effectively. However, acquiring high-quality pediatric data poses significant challenges, as pediatric datasets are often much smaller and harder to access compared to adult datasets. The cost of generating sufficient pediatric data can be prohibitively high, which often leads researchers to rely on public databases or collaborative networks to supplement the available data. To address the issue of data scarcity, several ML techniques can be employed to augment the dataset. For instance, transfer learning involves utilizing models pretrained on large adult datasets, which are then fine-tuned with pediatric data (Pisanello et al., 2019). This approach allows researchers to leverage existing knowledge, minimizing the need for extensive pediatric datasets. Data augmentation techniques, such as rotation, scaling, and cropping, can also be applied to generate additional data samples from existing datasets, thereby expanding the dataset size (Raghupathi and Raghupathi, 2014a). Additionally, synthetic data generation methods, like Generative Adversarial Networks (GANs), can be used to produce realistic, synthetic data to supplement real-world data (Frid-Adar et al., 2018).

Deep learning has also shown promise in predicting pediatric AMR, particularly in identifying complex patterns of pathogen resistance, such as in neonatal sepsis diagnosis (McAdams et al., 2022; Nguyen et al., 2019). However, deep learning models require high computational resources and significant optimization of both algorithms and hardware to operate efficiently. This computational complexity must be addressed to make deep learning models viable for pediatric AMR applications.

The collection and annotation of pediatric data can be more challenging than for adults, as children may have difficulty accurately expressing their symptoms, which can affect the completeness and accuracy of the data. To overcome this, several ML strategies can be employed. Multi-source data integration, which combines data from multiple medical institutions and diverse sources, can help increase dataset diversity and improve model generalization (Rajkomar et al., 2018). Semi-supervised and self-supervised learning techniques, which allow for the use of unannotated data, can enhance model performance by making better use of the available data (Sun et al., 2017; Van Engelen and Hoos, 2020). Active labeling, where medical experts manually annotate the most informative data, improves the quality and efficiency of the labeling process (He et al., 2019).

Moreover, pediatric pathogens and diseases possess unique characteristics that ML models must recognize in order to make accurate predictions. Feature engineering, specifically tailored to the pediatric context, is essential to ensure that the models capture these unique features (Grossman et al., 2017). Ensemble learning methods, which combine predictions from multiple models (e.g., decision trees, RF), have been shown to improve overall model accuracy and robustness (Dietterich, 2000). These approaches help ensure that models perform well even when faced with limited or noisy data.

In summary, common ML models used for predicting pediatric antibiotic susceptibility include Adaptive Boosting (AdaBoost), RF, Extreme Gradient Boosting (XGBoost), ensemble learning, SVM, and neural networks. The most suitable algorithm depends on the specific nature of the data and the goals of the study. While no single “best” algorithm exists for predicting drug susceptibility from sequencing data, many studies utilize a multi-algorithm approach to compare performance and identify the most effective model for their specific task (Hu et al., 2023; Xu Y. et al., 2024). Among these, RF has proven particularly effective in handling sequencing data focused on resistance genes, as it excels in capturing non-linear relationships and making predictions based on complex decision rules. This makes RF a promising candidate for predicting antimicrobial resistance in pediatric infections (Prasanna and Niranjan, 2019). A detailed overview of these algorithms’ performance is provided in Table 4.

**TABLE 4:** Utilizations of various supervised learning algorithms.

Algorithm	Year	Key advantages	Limitations	Typical application scenarios	Use in pediatric infectious disease	References
Neural networks	1958	1. Model flexibility 2. Powerful predictive capability 3. Handling a large number of features 4. Dealing with non-linear relationships	1. Sensitive to training data bias 2. Overfitting issue 3. High training complexity 4. Time-consuming 5. High model interpretability difficulty	1. Non-linear problem processing 2. High-dimensional data learning 3. Processing of unstructured data	ARGNet classify ARGs from sequence data, contributes significantly to understanding ARGs	Pei et al., 2024; López-Cortés et al., 2024
Decision tree	1959	1. Easy interpretation of results 2. Naturally handle missing values in data 3. Applicable to a broad range of complex problems 4. Minimal data preparation required	1. Pliable to overfit training data 2. Sensitive to data variations and noise 3. High computational complexity and cost	1. Non-linear data scenarios 2. Small data sets with relatively simple problems 3. Model interpretability required 4. Feature selection	Optimizing treatment outcomes and avoiding empirical medication use	Sick-Samuels et al., 2020; Costa and Pedreira, 2023
K-Nearest neighbors	1968	1. Simple and intuitive high accuracy 2. No training required 3. Suitable for multi-class problems 4. Supports multi-class classification	1. High computational cost 2. Large storage requirements 3. Poor performance with high-dimensional data	1. Small to medium scale datasets and data mining 2. Rapid prototyping design 3. Classification and regression problems	–	Pan et al., 2020
Linear regression	–	1. Easy to understand and interpret 2. Simple calculations, fast predictions 3. Broad applicability	1. Linearity assumption limitation 2. Sensitive to outliers 3. Multicollinearity affects model stability and interpretability 4. Independence assumption may not hold in practice	1. Continuous values 2. Economics and finance 3. Modeling simple relationships 4. Rapid prototyping and preliminary analysis 5. Scenarios with low data dimensions	Linear regression exhibits good applicability in analyzing trends of ARGs	Sivasankar et al., 2023; Maulud and Abdulazeez, 2020
Logistic regression	1972	1. Easy to understand and implement with fast speed 2. Clear explanation of model decision-making 3. Seamless extension to multi-class classification problems 4. Interpretable result weights	1. Limited by linear assumptions and multicollinearity issues 2. Prone to overfitting with high-dimensional data 3. Sensitive to outliers and requires a large sample size	1. Binary classification problems 2. High-dimensional sparse data scenarios 3. Medical diagnosis, marketing, credit scoring, etc.	1. Analysis of pathogen proportions isolated from pediatric urine samples and their resistance patterns to selected antibiotics 2. Identification of potential risk factors for clinical outcomes in MRSA and *S. aureus* infections	Bagnasco et al., 2022; Dai et al., 2024
Adaptive boosting	1995	1. Enhancing the performance of weak learners 2. Automatic adjustment of weak learner weights 3. Applicable for handling imbalanced class distributions	1. Sensitive to noise and outliers 2. Long training time	1. Binary classification, multi-class classification, and regression problems 2. Anomaly detection 3. Feature selection 4. Combination use with other ML methods	–	Feng et al., 2020
Support vector machine	1995	1. Effective in high-dimensional spaces and with small sample sizes 2. High memory efficiency 3. Multi-functionality and generalization capability 4. Robust to noise in data	1. Dependent on parameter selection 2. Potentially high computational cost, long training time 3. Potentially high computational cost, long training time	1. Highly effective in high-dimensional space 2. Large-scale datasets 3. Time series prediction scenarios 4. Bioinformatics analysis 5. Text classification, anomaly detection, etc.	–	Sunuwar and Azad, 2021; Cervantes et al., 2020
Random forests	2001	1. Possesses good robustness 2. Fast training speed and high accuracy 3. Broad applicability 4. Provides feature importance measures	1. High computational complexity, large memory usage, and long prediction time 2. Prediction results sometimes lack explainability 3. May overfit training data	1. Small samples, non-linear problems 2. Feature selection 3. Large-scale datasets, high-dimensional data 4. Time series prediction 5. Text classification and anomaly detection	–	Romandini et al., 2021
Extreme gradient boosting	2014	1. Applicable to large-scale datasets 2. Built-in regularization to prevent overfitting, high performance, and accuracy 3. Enhances model flexibility 4. Provides feature importance scores	1. Requires careful parameter tuning, risk of overfitting 2. Poor model interpretability 3. High memory consumption, potentially high computational Cost	1. Suitable for simple problems with low feature dimensions 2. Feature selection 3. Optimization algorithms 4. Large-scale datasets 5. Scenarios requiring high efficiency and accuracy in predictions	XGBoost possesses potential application value in pediatric infectious disease AMR research and clinical decision support systems	Xuemei et al., 2024; Huang et al., 2021

ARGs, antibiotic resistance genes; MRSA, methicillin-resistant *Staphylococcus aureus*; ML, machine learning.

### 6.3 Validation of ML model performance

The robustness and generalizability of ML models in pediatric AMR prediction must be thoroughly validated using authentic clinical data from diverse pediatric cohorts. This validation is essential for ensuring that models trained on one dataset can perform effectively across different settings, including those with distinct microbial populations and patient demographics. Consequently, regular validation against traditional surveillance data is imperative for assessing the robustness of these models (Lazer et al., 2014). Pediatric data is often scarce, making cross-validation and model robustness testing even more crucial to confirm the model’s applicability in clinical practice.

Different ML algorithms demonstrate variable predictive performance depending on their specific applications. To ensure comprehensive classification and representation of all MIC of antimicrobial drugs in the training set, stratified random sampling techniques—such as those implemented in ShuffleSplit software—are utilized. This method produces stratified, randomly collapsed datasets, thereby maintaining the proportionality of samples at each MIC level. Evaluating the predictive performance of ML models involves several key metrics, including Essential Agreement (EA), Category Agreement (CA), Receiver Operating Characteristic (ROC) curves, and AUC values. These metrics, alongside recall/sensitivity, specificity, Positive Predictive Value (PPV), Negative Predictive Value (NPV), Major Error (ME), and Very Major Error (VME), are critical for a comprehensive assessment of model efficacy (Shehadeh et al., 2021).

## 7 Future insights

The integration of ML into pediatric infectious disease management is still in its early stages, with the majority of current efforts primarily focusing on individual pathogens. While this has contributed to an initial understanding of pathogen-specific antibiotic resistance patterns, such an approach presents considerable limitations, particularly in the context of mixed infections. In clinical scenarios, pediatric patients-especially those in critical care-are frequently affected by polymicrobial infections, where multiple pathogens coexist. The challenge of accurately predicting antibiotic resistance in these complex infections is exacerbated by the technical difficulties of simultaneously identifying multiple pathogens. However, achieving this capability would be a significant breakthrough, as it could substantially improve clinical decision-making by providing a more comprehensive understanding of the pathogen landscape in each individual patient.

Currently, no gold-standard method exists to explore and predict the resistome-the collection of all resistance genes-within pediatric infections. The identification of AMR in children remains one of the most pressing challenges in modern medicine. The growing emergence of pediatric drug-resistant infections necessitates a concerted effort from both clinicians and researchers to develop more effective diagnostic tools and therapeutic strategies. While research on the application of ML to pediatric AMR is still limited, the increasing volume of studies in this field underscores its importance. Enhanced attention to this area is critical for improving the treatment of pediatric drug-resistant infections, mitigating the rise of AMR, and ultimately preventing further complications in vulnerable pediatric populations.

Advancements in WGS and NGS technologies have provided a powerful foundation for exploring antibiotic susceptibility, conducting evolutionary analyses, and uncovering the molecular mechanisms of AMR. These technologies have enabled more precise detection of resistance genes and their distribution across different microbial species, thereby facilitating a deeper understanding of AMR dynamics. As the number of studies utilizing NGS and ML continues to grow, clinical AMR surveillance has become more robust, offering clinicians valuable insights that can guide treatment decisions.

Particularly in pediatrics, where patients are at an increased risk for severe infections and acquired AMR, accurate predictions of drug susceptibility are crucial for improving clinical outcomes. The ability to rapidly and accurately assess the susceptibility of pathogens to specific antibiotics can directly inform therapeutic decisions, optimizing the use of antibiotics and reducing the risk of treatment failure.

Looking ahead, there is tremendous potential to develop integrated ML-driven platforms that not only detect antimicrobial resistance but also translate this information into personalized treatment recommendations for pediatric patients. Such platforms could combine pathogen resistance profiles with patient-specific data to create individualized treatment plans, ultimately streamlining clinical workflows and improving patient care. This would mark a major advancement in pediatric microbiology, providing clinicians with real-time, actionable data that would enhance the precision of diagnosis and the efficacy of treatments. These platforms could also be standardized for quality control, ensuring that clinical decisions are based on reliable, reproducible data.

**FIGURE 3:**
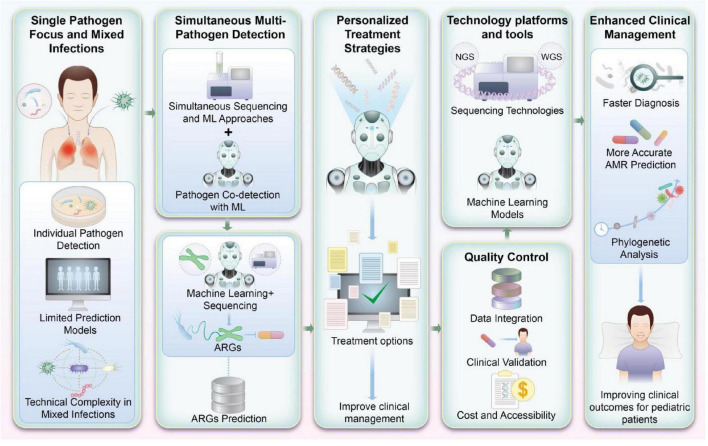
Machine learning (ML)-based integrated platform for detecting antibiotic susceptibility and antibiotic resistance genes (ARGs) in pediatrics. This figure visually encapsulates the prospective advancements in research and application for detecting antibiotic susceptibility and ARGs in pediatric infections. By integrating ML with high-throughput genomic sequencing, this approach not only enhances the accuracy of detecting multiple pathogens but also supports the refinement of antibiotic treatment strategies. Such advancements are pivotal for improving clinical management and therapeutic outcomes in pediatric patients.

As Figure 3 illustrates, ML-based platforms designed to detect antibiotic susceptibility and ARGs hold promise for transforming pediatric infectious disease management. Future research should focus on refining these systems, incorporating diverse pathogen profiles, and developing strategies for their seamless integration into routine clinical practice.

## 8 Conclusion

Advancements in sequencing technologies, such as mNGS, TS, and ONT, are meeting critical clinical needs in the detection and characterization of pathogens and resistance mechanisms. When integrated with ML, these methodologies have the potential to significantly improve the accuracy of predicting antimicrobial susceptibility, particularly for pediatric infections. This combination not only promises to enhance antibiotic stewardship but also offers the possibility of real-time, data-driven decision-making in clinical settings. As research progresses and these approaches are validated in clinical practice, they are expected to become integral tools for combating antimicrobial resistance in pediatric populations, ultimately transforming the management of infections in this vulnerable group.
